# Supplementary material for: Insulin-like 3 affects zebrafish spermatogenic cells directly and via Sertoli cells
Source: Commun Biol. 2021 Feb 15;4:204. doi: 10.1038/s42003-021-01708-y (PMC7884674; doi:10.1038/s42003-021-01708-y)
Supplement: Supplementary file 1 — Supplementary Information [file 42003_2021_1708_MOESM1_ESM.pdf]

Supplementary Information

Insulin-like 3 affects zebrafish spermatogenic cells directly and via Sertoli cells

Diego Crespo, Luiz H. C. Assis, Yu Ting Zhang, Diego Safian, Tomasz Furmanek, Kai Ove Skaftnesmo, Birgitta Norberg, Wei Ge, Yung-Ching Choi, Marjo J. den Broeder, Juliette Legler, Jan Bogerd, Rüdiger W. Schulz

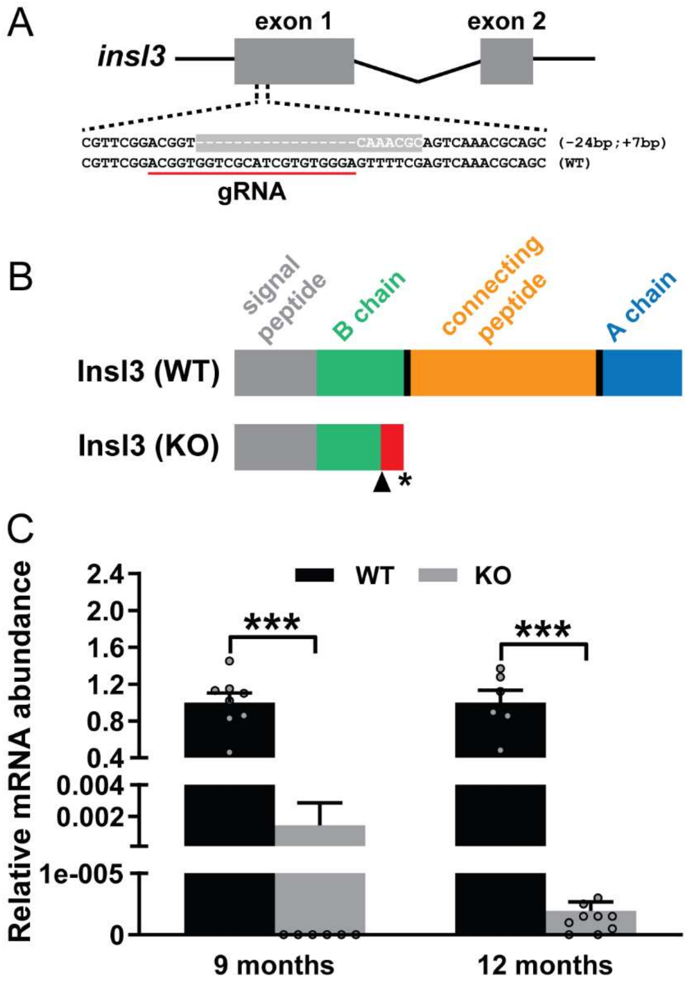

Supplementary Fig. 1 Efficient disruption of zebrafish *insl3* by CRISPR/Cas9. a Design of the guide RNA (gRNA) target site on exon 1 targeting the *insl3* gene (underlined sequence). Representative Sanger sequencing results showing the indels identified (highlighted with a grey background) in the *insl3* mutant line generated in this study. Numbers to the right of the mutant sequence indicate the loss or gain of bases, with the number of bases deleted (-) or inserted (+). WT, wild-type; bp, base pair. **b** Schematic diagram showing the functional domains of the WT Insl3 protein *versus* the *insl3* knockout (KO) mutant sequence. The black arrowhead indicates the start of the indel (the insertion indicated with the red box) and the asterisk indicates a premature stop codon caused by the frame shift. Black boxes in the Insl3 precursor indicate proteolytic processing sites. **c** CRISPR/Cas9-mediated decrease of *insl3* transcript levels in both 9 and 12 month-old zebrafish KO testes. Data are shown as mean fold change ± SEM (WT and KO, 9 months: N = 8 and 7; WT and KO, 12 months: N = 6 and 10; ***, $p < 0.001$) and expressed relative to the WT control group (which is set at 1).

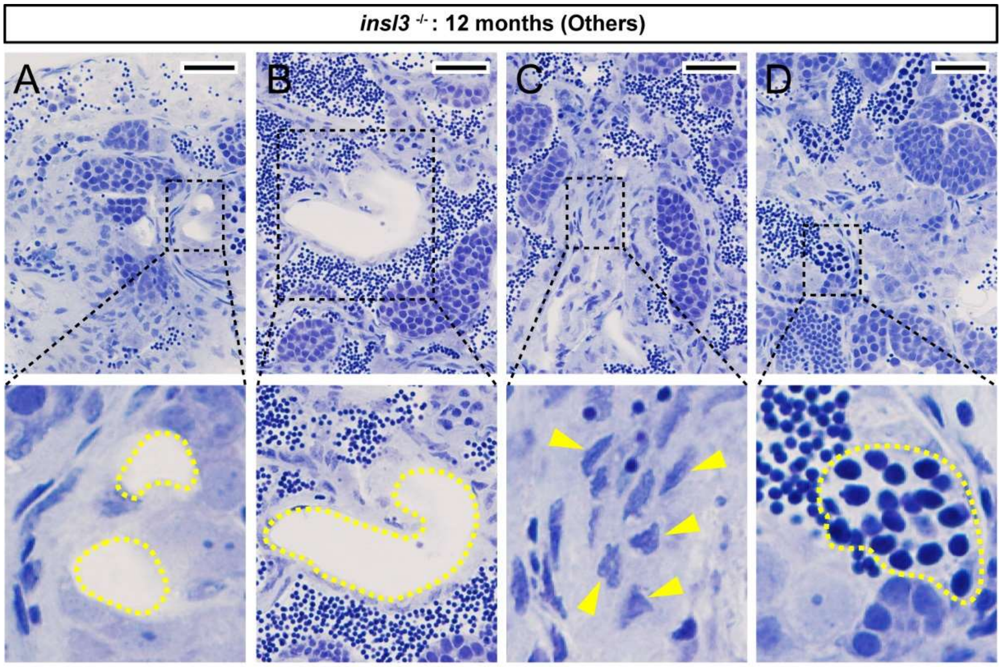

Supplementary Fig. 2 Composition of the category "Others" shown in Fig. 2d. a-d Areas found exclusively (**a**-**c**) or more prominently (**d**) in 12 months-old *insl3* knockout (*insl3*$^{-/-}$) testes. **a** Empty areas adjacent to Sertoli and/or germ cells within the germinal epithelium. **b** Empty areas lined by cytoplasmic extensions of Sertoli cells within the germinal epithelium. **c** Areas containing an accumulation of Sertoli cells. **d** Areas containing apoptotic germ cells. The stippled black areas identify the magnified areas. The stippled yellow lines in **a** indicate empty space lined by germ and/or Sertoli cells, or lined only by Sertoli cell extensions in **b**, in both cases not forming part of the tubular lumen. Yellow arrowheads in **c** indicate areas showing an accumulation of only Sertoli cells. The yellow stippled line in **d** indicates a group of germ cells (type B spermatogonia) that show the typical appearance of apoptotic cells in toluidine-blue stained plastic sections (shrinkage, losing at least partially the contact with the immediate tissue environment, pyknosis/nuclear fragmentation[3]). Scale bars, 25 µm.

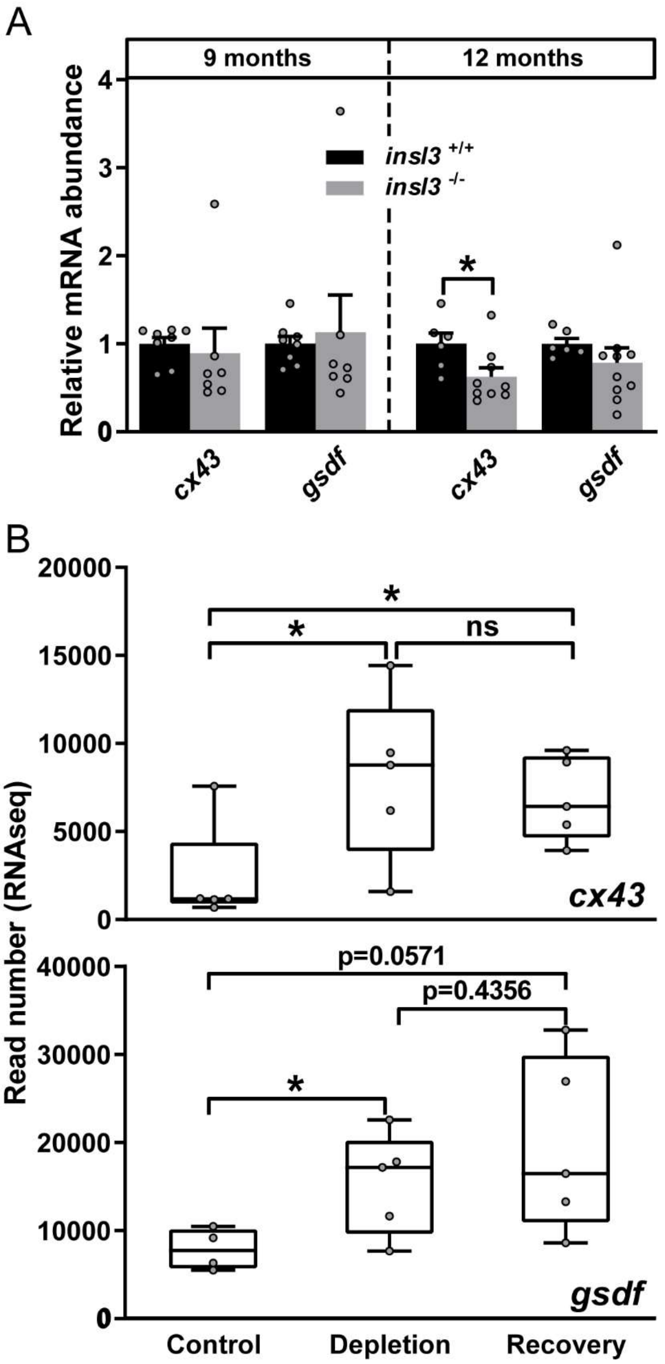

Supplementary Fig. 3 Testicular expression of selected Sertoli cell genes in *insl3* knockout mutant gonads, and in control, depleted and recovering gonads. a *cx43* and *gsdf* transcript levels in wild-type (*insl3*$^{+/+}$) and *insl3* knockout (*insl3*$^{-/-}$) testes 9 and 12 months post-fertilization. Data are shown as mean fold change ± SEM (*insl3*$^{+/+}$ and *insl3*$^{-/-}$, 9 months: N = 8 and 7; *insl3*$^{+/+}$ and *insl3*$^{-/-}$, 12 months: N = 6 and 10; *, $p < 0.05$) and expressed relative to the wild-type control group (which is set at 1). **b** Expression levels of *cx43* and *gsdf* in control, germ cell-depleted (by exposure to the cytostatic agent busulfan[1]), and testes with recovering (from busulfan) spermatogenesis, as described by Crespo et al.[2] (NCBI GEO data set GSE116611). Data are expressed as mean ± SEM (N = 5; *, $p < 0.05$). ns, not significant differences between groups.

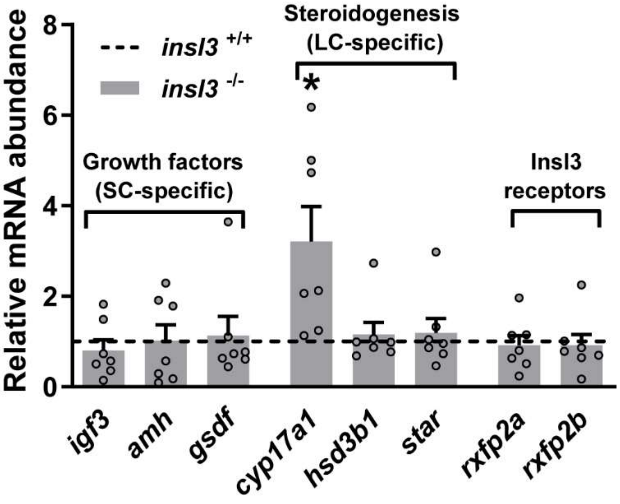

Supplementary Fig. 4 Testicular expression of selected genes in 9 month-old *insl3* CRISPR-ed zebrafish. Transcript levels of growth factors, steroidogenesis-related and Insl3 receptors in 9 month-old wild-type (*insl3*$^{+/+}$) and *insl3* knockouts (*insl3*$^{-/-}$) adult testis tissue. Data are mean fold change ± SEM (*insl3*$^{+/+}$ and *insl3*$^{-/-}$, N = 8 and 7; * $p < 0.05$) and expressed as relative to the wild-type group (which is set at 1; dashed line). SC, Sertoli cell; LC, Leydig cell.

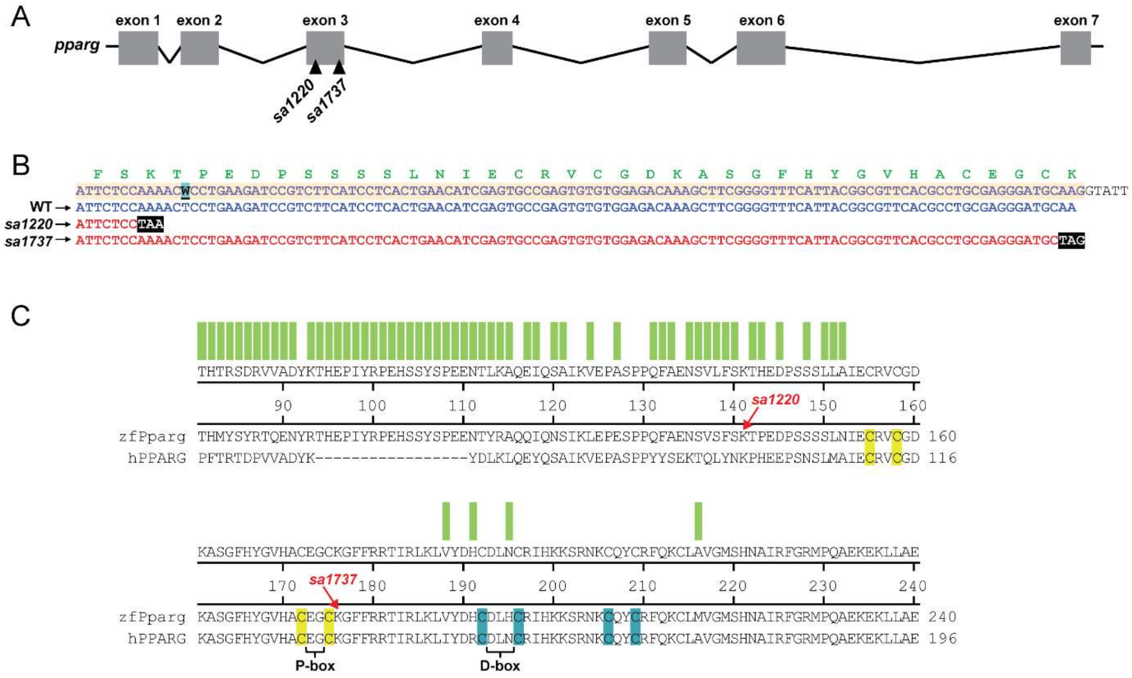

Supplementary Fig. 5 Schematic description of the zebrafish mutant lines for *pparg* obtained from the Zebrafish International Resource Center (ZIRC). a Diagram showing both point mutations (*sa1220* and *sa1737*) in exon 3 of the *pparg* gene, as indicated by the black arrowheads. **b** Alignment showing the wild-type (WT) *pparg* nucleotide sequence *versus* the *pparg* knockout mutant sequences. Both mutant *pparg* alleles have an A>T nonsense mutation leading to a premature stop codon (black background) at amino acid 141 ($pparg^{-/-\ sa1220}$) or amino acid 176 ($pparg^{-/-\ sa1737}$). **c** Amino acid alignment of human and zebrafish PPARG/Pparg proteins, showing amino acid disagreement (green bars), the cysteine residues constituting zinc finger 1 (yellow) and zinc finger 2 (cyan). The Pparg protein in mutant *sa1220* lacks both zinc fingers of the DNA-binding domain as well as the ligand-binding domain (LBD), while the Pparg protein in mutant *sa1737* retains the first zinc finger, including the P-box (responsible for sequence-specific DNA recognition), but lacks the second zinc finger, which harbours the D-box (involved in protein-protein cooperative interaction in the dimerization process) and the subsequent LBD.

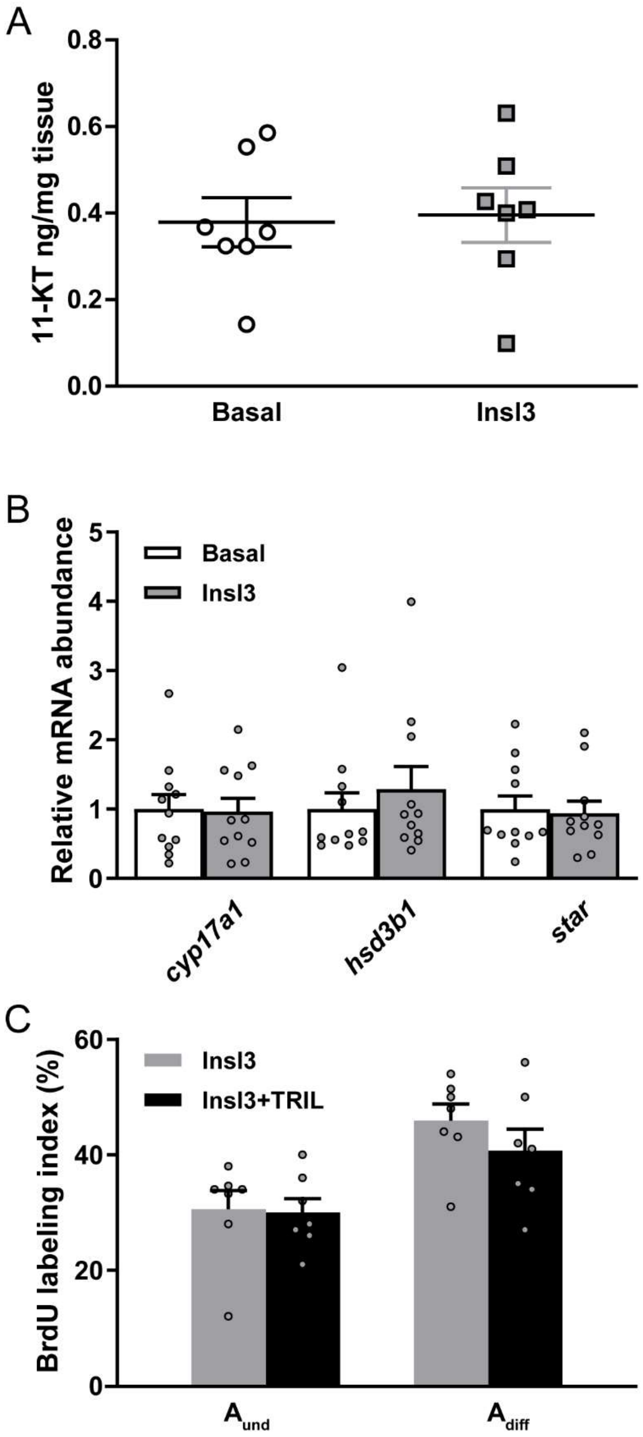

Supplementary Fig. 6 Insl3-induced effects on spermatogenesis are steroid-independent in zebrafish. a-b 11-ketotestosterone (11-KT) levels in medium (**a**) and steroid-related gene expression in testis tissue (**b**) after incubation in the absence or presence of 100 ng/mL Insl3 for 4 days. **c** Evaluation of the proliferation activity of type A spermatogonia in zebrafish testes cultured for 4 days with 100 ng/mL Insl3, and in the absence or presence of trilostane (TRIL; 25 µg/mL). In **a** and **c**, data are shown as mean ± SEM (N = 7), and in **b** as mean fold change ± SEM (N = 11) and expressed relative to the basal control group (which is set at 1).

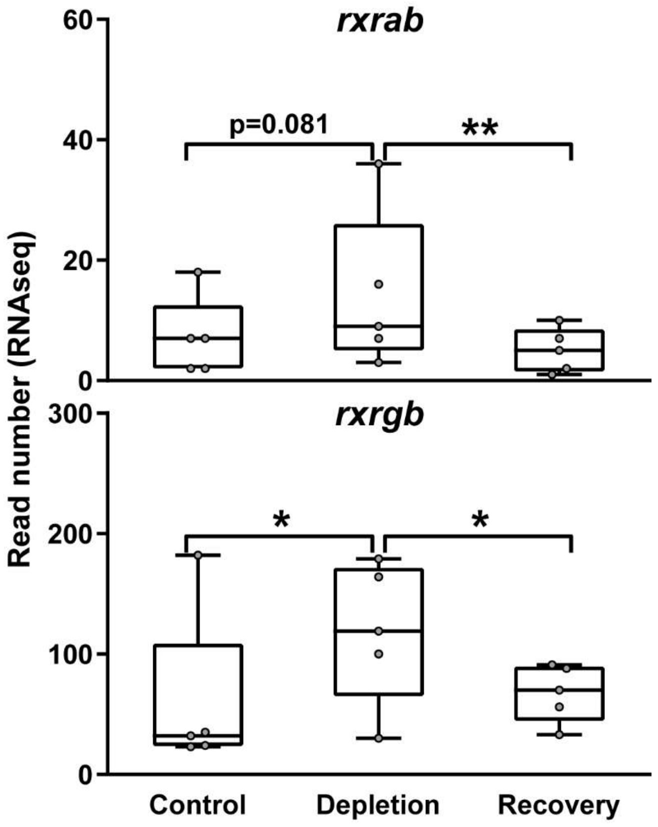

Supplementary Fig. 7 Testicular expression of selected retinoid X receptors in control, depleted and recovering gonads. Expression levels of *rxrab* and *rxrgb* in control, germ cell-depleted (by exposure to the cytostatic agent busulfan[1]), and testes with recovering (from busulfan) spermatogenesis, as described by Crespo et al.[2] (NCBI GEO data set GSE116611). Data are expressed as mean ± SEM (N = 5; *, $p < 0.05$; *, $p < 0.01$).

Supplementary Table 1 Sequences of primers used for the generation of transgenic lines.

Transgenic line	Primers	Primer sequence (5´→ 3')
Tg(rxfp2a:EGFP): zebrafish *rxfp2a* promoter (3193 bp, preceding the ATG start codon)	4703 (Fw)<br>4704 (Rv)	TAGGCCGGCCTATCACTGAGCTTTATCTAAAGATTTTTATAAT<br>TTCGGCGCGCCAGTGAAGGCTTTACAAAAGCATGACCGA
Tg(rxfp2b:mCherry): zebrafish *rxfp2b* promoter (3087 bp, preceding the ATG start codon)	4705 (Fw)<br>4706 (Rv)	TAGGCCGGCCTCATAATTAAACCTACCGTTATGAAACTGAAGTACC<br>TTCGGCGCGCCTGTGGCGTCGTGCCAGTCATGC

Fse I restriction enzyme site; Asc I restriction enzyme site.

Supplementary Table 2 Sequences of primers used in gene expression analyses by qPCR.

Target gene	Gene description	Primers	Primer sequence (5′→ 3')
aldh1a2	aldehyde dehydrogenase 1 family, member A2	4359 (Fw)<br>4360 (Rv)	CGCTGGATGGGCAGATAAGA<br>TCTGGTGAGGGTGAAAAATTCTC
amh	anti-Mullerian hormone	4486 (Fw)<br>4487 (Rv)	CTCTGACCTTGATGAGCCTCATTT<br>GGATGTCCCTTAAGAACTTTTGCA
casp9	caspase 9, apoptosis-related cysteine peptidase	6169 (Fw)<br>6170 (Rv)	ACATACGACTGCTGTGTGGTCAT<br>GAACTGCACCAGGGAATCGA
cyp17a1	cytochrome P450, family 17, subfamily A, polypeptide 1	2773 (Fw)<br>2774 (Rv)	GGGAGGCCACGGACTGTTA<br>CCATGTGGAACTGTAGTCAGCAA
cyp26a1	cytochrome P450, family 26, subfamily A, polypeptide 1	4383 (Fw)<br>4384 (Rv)	TGGGCTTGCCGTTCATTG<br>CATGCGCAGAAACTTCCTTCTC
eef1a1l1	eukaryotic translation elongation factor 1 alpha 1, like 1	2476 (Fw)<br>2477 (Rv)	GCCGTCCCACCGACAAG<br>CCACACGACCCACAGGTACAG
gsdf	gonadal somatic cell derived factor	2366 (Fw)<br>2367 (Rv)	CATCTGCGGGAGTCATTGAAA<br>CAGAGTCCTCCGGCAAGCT
hsd3b1	hydroxy-delta-5-steroid dehydrogenase, 3 beta- and steroid delta-isomerase 1	5096 (Fw)<br>5097 (Rv)	GATCCGACTGCTGGATAGAAACA<br>CCCGGCAATCATCAAGAGA
insl3	insulin-like 3 (Leydig cell)	2466 (Fw)<br>2467 (Rv)	TCGCATCGTGTGGGAGTTT<br>TGCACAACGAGGTCTCTATCCA
rpl13a	ribosomal protein L13a	3998 (Fw)<br>3999 (Rv)	GAGCCCCCAGCAGAATCTTC<br>AGCCTGACCCCTCTTGGTTTT
rxfp2a	relaxin family peptide receptor 2a	3258 (Fw)<br>3259 (Rv)	CAATTCCAGTCTCTGTCAGCACAT<br>CTCAACGTCATTCTCCGCAAA
rxfp2b	relaxin family peptide receptor 2b	3262 (Fw)<br>3263 (Rv)	CTGCCAGACTCTGTGCCCATA<br>AGTCGTGATGCTATTACCCTCGAA
star	steroidogenic acute regulatory protein	2546 (Fw)<br>2547 (Rv)	CCTGGAATGCCTGAGCAGAA<br>ATCTGCACTTGGTCGCATGAC
ubc	ubiquitin C	3951 (Fw)<br>3952 (Rv)	CCATACACCGCACTCTTACAGAAA<br>CCAGTCAGCGTCTTCACAAAGAT
xiap	X-linked inhibitor of apoptosis	6159 (Fw)<br>6160 (Rv)	CATCCCATTGACCCTGAGAGA<br>CCTCCACATCTGAAGCACATCA

Fw, forward; Rv, reverse.

References

1 Nobrega, R. H. *et al.* Spermatogonial stem cell niche and spermatogonial stem cell transplantation in zebrafish. *PLoS One* **5**, doi:10.1371/journal.pone.0012808 (2010).

2 Crespo, D. *et al.* Endocrine and local signaling interact to regulate spermatogenesis in zebrafish: follicle-stimulating hormone, retinoic acid and androgens. *Development* **146**, doi:10.1242/dev.178665 (2019).

3 Elmore, S. Apoptosis: a review of programmed cell death. *Toxicol Pathol* **35**, 495-516, doi:10.1080/01926230701320337 (2007).
