# Supplementary material for: Insulin-like 3 affects zebrafish spermatogenic cells directly and via Sertoli cells
Source: Commun Biol. 2021 Feb 15;4:204. doi: 10.1038/s42003-021-01708-y (PMC7884674; doi:10.1038/s42003-021-01708-y)
Supplement: Supplementary file 4 — Description of Additional Supplementary Files [file 42003_2021_1708_MOESM4_ESM.pdf]

Description of Additional Supplementary Files

File name: Supplementary Data 1.

Description: Mapping statistics and complete list of differentially expressed genes identified by RNAseq ($N = 3$; $p < 0.05$) in Insl3-treated testis tissue.

File name: Supplementary Data 2.

Description: Source data underlying the graphs shown in the manuscript and Supplementary information.
