# Supplementary material for: Insulin-like 3 affects zebrafish spermatogenic cells directly and via Sertoli cells
Source: Commun Biol. 2021 Feb 15;4:204. doi: 10.1038/s42003-021-01708-y (PMC7884674; doi:10.1038/s42003-021-01708-y)
Supplement: Supplementary file 6 — Reporting Summary [file 42003_2021_1708_MOESM6_ESM.pdf]

nature research

Corresponding author(s): Rüdiger W. Schulz

Last updated by author(s): Jan 7, 2021

Reporting Summary

Nature Research wishes to improve the reproducibility of the work that we publish. This form provides structure for consistency and transparency in reporting. For further information on Nature Research policies, see our Editorial Policies and the Editorial Policy Checklist.

Statistics

For all statistical analyses, confirm that the following items are present in the figure legend, table legend, main text, or Methods section.

n/a	Confirmed	
☐	☒	The exact sample size (*n*) for each experimental group/condition, given as a discrete number and unit of measurement
☐	☒	A statement on whether measurements were taken from distinct samples or whether the same sample was measured repeatedly
☐	☒	The statistical test(s) used AND whether they are one- or two-sided<br>*Only common tests should be described solely by name; describe more complex techniques in the Methods section.*
☒	☐	A description of all covariates tested
☐	☒	A description of any assumptions or corrections, such as tests of normality and adjustment for multiple comparisons
☐	☒	A full description of the statistical parameters including central tendency (e.g. means) or other basic estimates (e.g. regression coefficient) AND variation (e.g. standard deviation) or associated estimates of uncertainty (e.g. confidence intervals)
☒	☐	For null hypothesis testing, the test statistic (e.g. *F*, *t*, *r*) with confidence intervals, effect sizes, degrees of freedom and *P* value noted<br>*Give P values as exact values whenever suitable.*
☒	☐	For Bayesian analysis, information on the choice of priors and Markov chain Monte Carlo settings
☒	☐	For hierarchical and complex designs, identification of the appropriate level for tests and full reporting of outcomes
☒	☐	Estimates of effect sizes (e.g. Cohen's *d*, Pearson's *r*), indicating how they were calculated

Our web collection on statistics for biologists contains articles on many of the points above.

Software and code

Policy information about availability of computer code

Data collection	CRISPR/Cas9 targets for the zebrafish insl3 gene were selected using ZiFiT Targeter software (http://zifit.partners.org/ZiFiT). DAVID Bioinformatics Resources 6.7 (http://david.ncifcrf.gov/) was used to retrieve Gene Ontology terms from the list of differentially expressed genes and exported as the input for each functional enrichment analysis.
Data analysis	Functional enrichment analyses were carried out using a plugin available at http://www.baderlab.org/Software/EnrichmentMap/ for the Cytoscape network environment. The human Protein Atlas database was used to retrieve information on PPARG/Pparg expression in testis tissue (https://www.proteinatlas.org/ENSG00000132170-PPARG/tissue/testis).

For manuscripts utilizing custom algorithms or software that are central to the research but not yet described in published literature, software must be made available to editors and reviewers. We strongly encourage code deposition in a community repository (e.g. GitHub). See the Nature Research guidelines for submitting code & software for further information.

Data

Policy information about availability of data

All manuscripts must include a data availability statement. This statement should provide the following information, where applicable:

- Accession codes, unique identifiers, or web links for publicly available datasets
- A list of figures that have associated raw data
- A description of any restrictions on data availability

The complete raw RNAseq data of the 6 samples sequenced in this study (3 biological replicates per condition) have been deposited in the NCBI GEO database under the accession number GSE152038 (data will become public in June 8, 2021). Results associated to this RNAseq data set are shown in Fig. 4 and Supplementary Data 1. Expression levels of selected genes in control, germ cell-depleted, and testes with recovering spermatogenesis (citation 25 of the submitted manuscript; Crespo et al., 2019) were retrieved using the GEO data set GSE116611. Results associated to this RNAseq data set are shown in Figs. 1b, 5e, and 6d,

Supplementary Figs. 3b and 7. All data generated or analyzed during this study are included in the submitted article (and its Supplementary information files). The source data underlying the graphs are provided in Supplementary Data 1 and 2.

Field-specific reporting

Please select the one below that is the best fit for your research. If you are not sure, read the appropriate sections before making your selection.

[x] Life sciences [ ] Behavioural & social sciences [ ] Ecological, evolutionary & environmental sciences

For a reference copy of the document with all sections, see nature.com/documents/nr-reporting-summary-flat.pdf

Life sciences study design

All studies must disclose on these points even when the disclosure is negative.

Sample size	No statistical method was applied to predetermine sample size. The number of wild type, mutant and transgenic zebrafish males used was based on experimental considerations and also on the variation observed in previous experiments of the same kind. For each experiment, sample size is indicated in the figure legend and reflects the number of independent biological and/or technical replicates.
Data exclusions	Outlier analysis of numerical values was performed by Grubb's test and outliers were excluded from calculations.
Replication	For ex vivo analyses (tissue culture experiments), 6-10 wild type males (biological replicates) were selected and analyzed in 2-3 technical replicates per biological replicate. Between 3 and 4 transgenic males were analyzed to investigate the cellular expression of Insl3 receptors 2a and 2b. In the ligand-receptor binding assays, 3 biological replicates (independent transfections) were carried out, and each data point was analyzed in triplicate (technical replicates). For histological, germ cell apoptosis and gene expression analyses of insl3 knockout testes, between 4 and 15 males were used. Between 3 and 4 pparg mutant males were analyzed for quantification of germ cell types. This information is included in the figure legends. All attempts of replication were successful.
Randomization	Due to the nature of our experiments, we did not use group allocation.
Blinding	All histological/morphological analyses were carried out blinded: persons carrying out the analyses did so working with coded slides. Sample identity was revealed after completing analysis of the slides/sections for statistical evaluation. In all qPCR analyses, samples were analyzed after having received randomized code numbers and sample identity was revealed after completion of the analyses for statistical evaluation.

Reporting for specific materials, systems and methods

We require information from authors about some types of materials, experimental systems and methods used in many studies. Here, indicate whether each material, system or method listed is relevant to your study. If you are not sure if a list item applies to your research, read the appropriate section before selecting a response.

Materials & experimental systems

n/a	Involved in the study	
[ ]	[x]	Antibodies
[ ]	[x]	Eukaryotic cell lines
[x]	[ ]	Palaeontology and archaeology
[ ]	[x]	Animals and other organisms
[x]	[ ]	Human research participants
[x]	[ ]	Clinical data
[x]	[ ]	Dual use research of concern

Methods

n/a	Involved in the study	
[x]	[ ]	ChIP-seq
[x]	[ ]	Flow cytometry
[x]	[ ]	MRI-based neuroimaging

Antibodies

Antibodies used	Purified mouse anti-BrdU (BD Biosciences). Clone B44 (RUO [GMP]). Catalog number 347580. Lot number 8151735.
Validation	BrdU incorporation was detected following a protocol modified from Van de Kant and de Rooij (Histochem J 1992; 24:170-175). Zebrafish testes were fixed for 5 h at room temperature in freshly prepared methacarn (60% [v/v] absolute ethanol, 30% chloroform, and 10% glacial acetic acid) and embedded in Technovit 7100 (Heraeus Kulzer). Five-micrometer-thick sections were subjected to antigen retrieval (1% [v/v] periodic acid in water at 60°C for 30 min) and peroxidase blocking (1% [v/v] H2O2 in PBS for 10 min). Thereafter, slides were incubated at room temperature for 1 h with mouse anti-BrdU (1:80 [BD Biosciences] diluted in PBS containing 1% [w/v] bovine serum albumin [BSA; Sigma-Aldrich]), and then for an additional hour with biotinylated horse anti-mouse (1:100 [Vector Laboratories] diluted in PBS containing 1% [w/v] BSA). Revelation of immunostaining was done using avidin-biotin complex incubation for 1 h (Vector Laboratories) followed by diaminobenzidine (Dako)<br>substrate development for 20 sec. Nuclei were counterstained with hematoxylin Gill no. 3 (Sigma-Aldrich) for 30 sec. For a negative control, the primary antibody (mouse anti-BrdU) was replaced with the same concentration of normal mouse immunoglobulin G (BD Biosciences).<br>Relevant citations and additional information can be found in the manufacture's website: https://www.bdbiosciences.com/us/

applications/research/apoptosis/purified-antibodies/purified-mouse-anti-brdu-b44/p/347580

Eukaryotic cell lines

Policy information about cell lines

Cell line source(s)	HEK293T cell line (from Hubrecht Institute, Uppsalalaan 8, 3584 CT Utrecht, The Netherlands).
Authentication	We did not authenticate the cell line after having received it.
Mycoplasma contamination	The cell line was negative for mycoplasma contamination when tested 3 months before the experiments described in the present MS.
Commonly misidentified lines (See ICLAC register)	n/a.

Animals and other organisms

Policy information about studies involving animals; ARRIVE guidelines recommended for reporting animal research

Laboratory animals	Zebrafish were bred and raised in the aquarium facility of the Faculty of Science at Utrecht University (The Netherlands). Sexually mature males between 3 and 12 months of age were used for the present experiments.
Wild animals	This study did not involve wild animals.
Field-collected samples	This study did not involve samples collected from the field.
Ethics oversight	Handling and experimentation were consistent with the Dutch national regulations. The Life Science Faculties Committee for Animal Care and Use in Utrecht approved the experimental protocols.

Note that full information on the approval of the study protocol must also be provided in the manuscript.
